# Microelectrode array measurements of fungal mycelium reveal growth-associated electrical activity

**DOI:** 10.1557/s43579-026-01041-7

**Published:** 2026-09-28

**Authors:** Mark Ablonczy, Sourav Patranabish, Sarah Schyck, Saurav Sharma, Hande Aydoğmus-Sasse, Vasso Giagka, Clementine Boutry, Kunal Masania

**Affiliations:** 1 Shaping Matter Lab, Faculty of Aerospace Engineering, Delft University of Technology, Kluyverweg 1, 2629 HS Delft, Netherlands; 2 Electronic Components, Technology and Materials (ECTM), Department of Microelectronics, Faculty of Electrical Engineering, Mathematics and Computer Science, Delft University of Technology, Mekelweg 4, 2628 CD Delft, Netherlands; 3 Bioelectronics, Department of Microelectronics, Faculty of Electrical Engineering, Mathematics and Computer Science, Delft University of Technology, Mekelweg 4, 2628 CD Delft, Netherlands

**Keywords:** Bioelectronic, Biomaterial, Microelectronics, Responsive, Sensor

## Abstract

**Abstract:**

Fungal bioelectricity has promising potential for biosensing in engineered living materials, but its nature remains unclear. We use a custom microelectrode array to record the extracellular electrical activity of mycelium, along with optical tracking and impedance measurements. Measurements across three mycelial growth experiments, along with a sterile control and biocide treatment, show that spontaneous signals coincide with mycelial growth. The signals contained transient spikes with rate and amplitude distributions dependent on growth. The measurements revealed distinct rise-and-fall patterns of electrical activity as mycelium overgrew the electrodes. These results establish an initial basis for future studies and living-material applications.

**Graphical abstract:**

**Figure:**
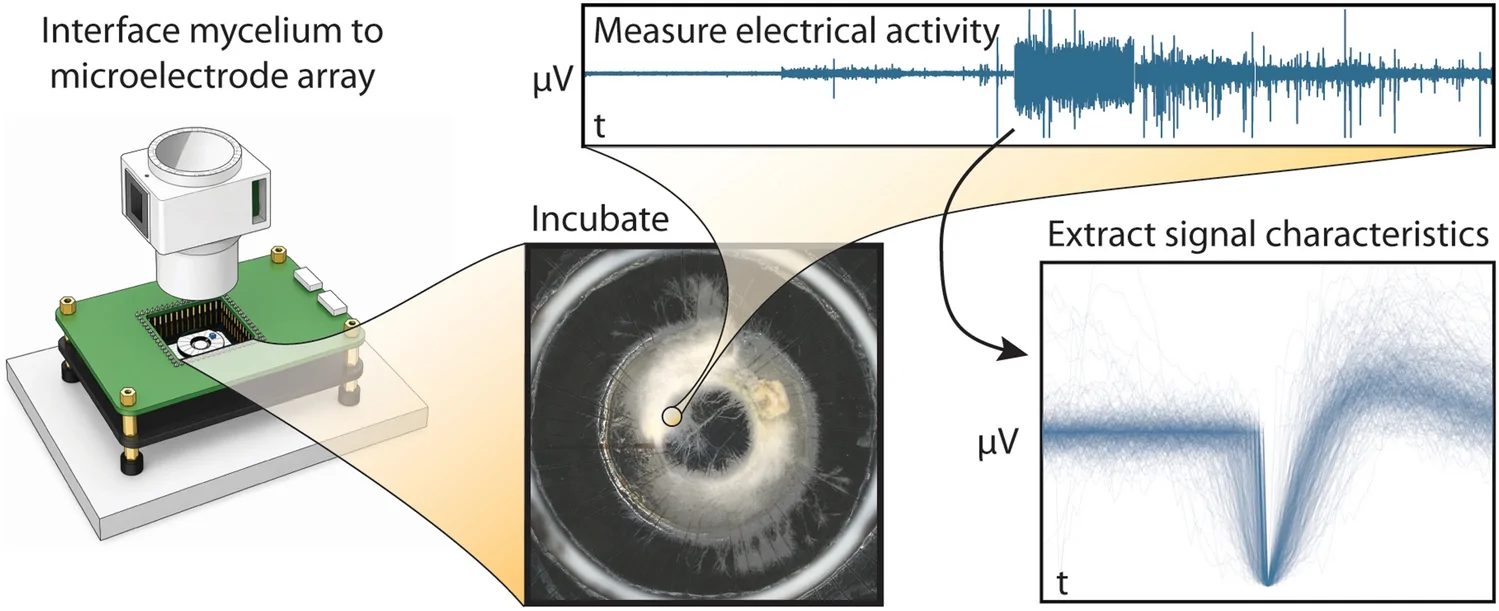

**Supplementary Information:**

The online version contains supplementary material available at https://doi.org/10.1557/s43579-026-01041-7.

## Introduction

The hallmarks of living systems include the ability to continuously sense, respond to, and adapt to their surroundings.^[1]^ Translating these capabilities into engineered materials could enable structures that monitor their own state, respond autonomously to stimuli, and adapt to changing environments.^[2]^ Such adaptive materials could contribute to sustainability by reducing the need for external sensing hardware, enabling longer material lifetimes, and supporting more efficient resource use. This possibility has driven growing interest in engineered living materials, an emerging class of systems in which living organisms are incorporated directly into materials as functional biosensing elements.^[3–5]^

Fungi are especially attractive candidates for such living-material systems. Mycelium, the filamentous growth form of fungi, can infiltrate porous substrates, grow in variable environments, and form connected networks across large areas.^[6]^ Recent work has demonstrated the feasibility of mycelium-based living materials and has suggested that fungal electrical activity could be used for sensing and control applications.^[7–10]^ For example, electrophysiological measurements from growing mycelium have been used to mediate sensorimotor control in soft robotic systems.^[8]^ Despite this promise, the practical use of fungal bioelectricity remains limited by an incomplete understanding of the measured signals, their dependence on growth state and environment, and the extent to which they can be localised to specific regions of developing mycelium.

A central unresolved issue is how fungal bioelectric activity should be interpreted. Several studies have reported spontaneous extracellular voltage fluctuations in fungal mycelia and have described them as action potential-like because their waveforms resemble the rise-and-fall dynamics of excitable biological signals.^[8,9,11,12]^ However, the interpretation of these signals and the terminology used to describe them remain contentious. Critics have noted that extracellular measurements made with large needle electrodes can be difficult to localise and may be vulnerable to non-biological contributions, such as electrode–medium interactions, Donnan potentials (electric potentials due to unequal distributions of ions in a charged polymer), vibration-induced artefacts, and electrochemical drift.^[13,14]^ More recent studies have made the biological interpretation more plausible by showing reduced electrical activity after treatment with cycloheximide,^[15]^ chloroform,^[16]^ and nystatin.^[17]^ However, these measurements still leave several questions unresolved: where the spikes originate, how they relate to local growth and morphology, and how much the recorded signal depends on electrode coupling or measurement artefacts. Spatially resolved recordings are therefore needed to separate local fungal activity from non-local or non-biological signals.

**Figure 1:**
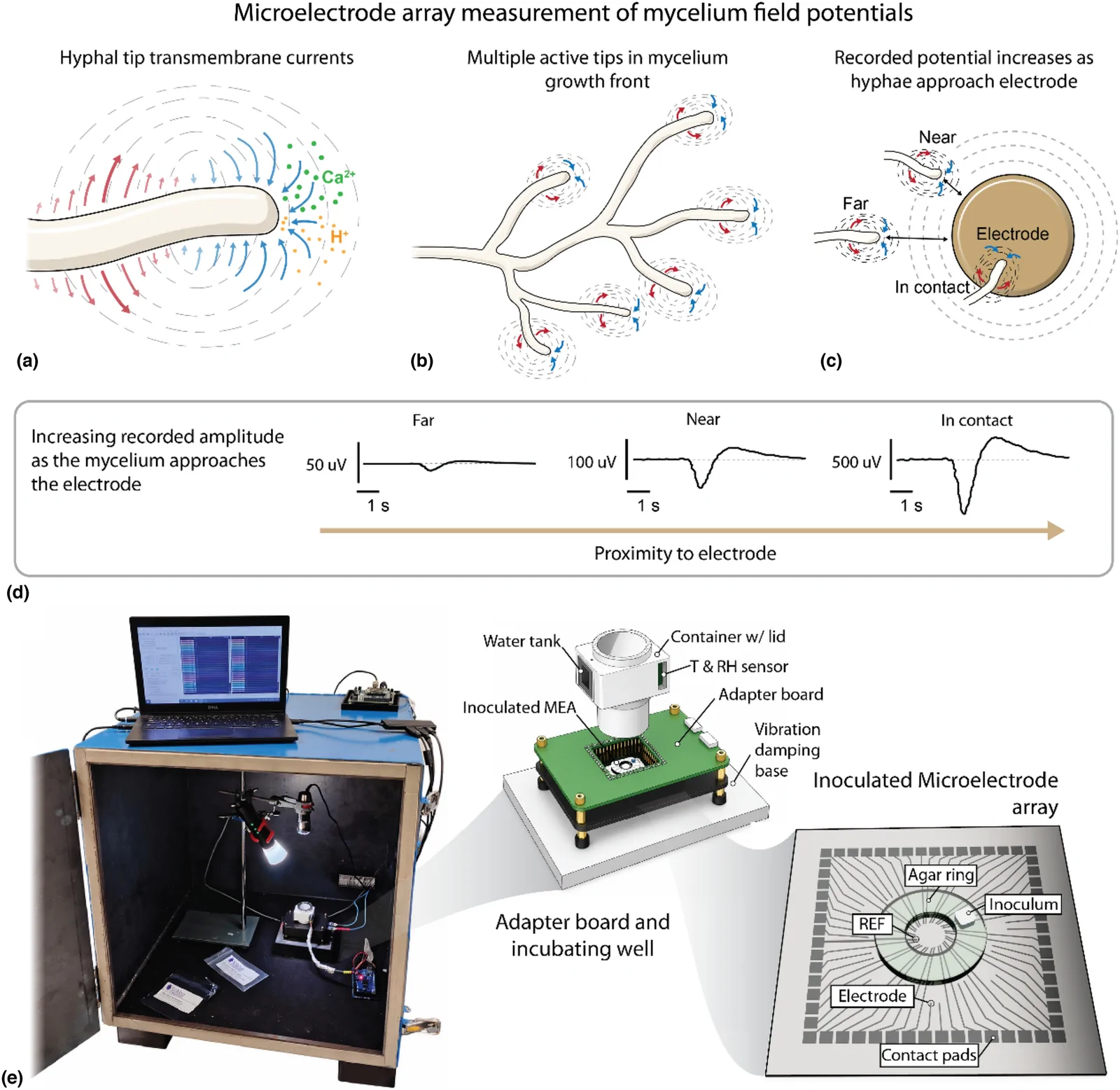
Overview of fungal bioelectric signal and MEA measurement. (a) Electric currents (blue in, red out) around a growing hypha, originating from the flow of protons and ions across the growing tip, adapted from Gow.^[18]^ (b) Illustration of a growing mycelial network in which actively growing hyphal tips may contribute local extracellular electrical activity. (c) Mycelium signalling measured with a microelectrode, where each growing tip produces electric field fluctuations that are sensed by the nearby electrode. (d) The recorded voltage signals are sensed by a microelectrode, with amplitude relating to proximity. (e) The MEA measurement experimental setup, depicting the Faraday shield and its contents, the adapter board and incubating well, and the inoculated microelectrode array.

There are plausible biophysical reasons to expect electrical activity near actively growing regions of fungal mycelium. Mycelium consists of branching microscopic filaments called hyphae, which extend primarily at their tips during growth. Earlier measurements in filamentous fungi showed steady transmembrane currents around growing hyphal tips, with positive current entering at the hyphal apex and leaving behind the tip [Fig. 1(a)], while non-growing hyphae produced little or no current.^[18]^ In a mycelial colony, multiple growing hyphal tips may individually produce transmembrane currents near the advancing growth front [Fig. 1(b)]. Such currents are generally discussed in relation to ion transport across the fungal membrane, including contributions from H^+^, Ca^2+^, and other ions involved in polarised growth and stress responses.^[3,19–21]^ Local fluctuations in these ion currents could generate extracellular voltage spikes near growing hyphal tips. As growing hyphae approach or contact an electrode [Fig. 1(c)], these extracellular voltage spikes would be expected to be recorded with increasing amplitude, depending on coupling, distance, and the conductive path to the reference [Fig. 1(d)]. The signal measured by an electrode would be expected to depend on the number of active tips nearby, their distance from the electrode, the local conductive path to the reference, and the evolving structure of the mycelial network.

Measuring fungal bioelectricity requires distinguishing local biological activity from signals arising from non-local colony-scale activity, electrode–medium interactions, or environmental artefacts. Needle electrode recordings have provided important evidence of low-frequency (< 10 Hz) electrical activity in fungal mycelia, but their large size limits source localisation.^[8,9,22]^ Microelectrode arrays offer a route to overcome this limitation by recording extracellular potentials across many spatially distributed microelectrodes.^[12,23]^ Recent MEA measurements of fungi have begun to analyse the spatial progression of low-frequency potentials in mycelium grown in liquid culture.^[17]^ Building on these advances, spatially resolved recordings during solid-substrate colonisation could further clarify how spontaneous extracellular voltage spikes relate to the advancing growth front, local mycelial morphology, and the development of the mycelial network.

To address this, we recorded spontaneous extracellular potentials from *Ganoderma lucidum* mycelium as it grew across a custom microelectrode array placed inside a controlled, shielded measurement setup [Fig. 1(e)]. The array contained 59 spatially distributed recording electrodes arranged in concentric regions around the centre, allowing electrical activity to be compared across different parts of the developing colony. Long-duration electrophysiological recordings were combined with optical tracking of colony growth, impedance measurements before, during, and after colonisation, and time- and frequency-domain signal analyses. This approach allowed us to relate extracellular electrical activity to the position of the advancing growth front and to local differences in mycelial coverage.

In our experiments, we observed that spontaneous extracellular voltage spikes are closely associated with mycelial growth. Electrical activity appeared as the colony advanced across the array, varied between regions with different degrees of mycelial coverage, and changed over time as the growth front passed individual electrodes. The resulting signals exhibited recurring features in their amplitude, frequency content, and spike waveform, considering the variability expected from a growing biological network. These measurements provide a spatially resolved example of growth-associated fungal electrical activity and may help guide future mechanistic studies and living-material sensing applications.

## Results and discussion

**Figure 2:**
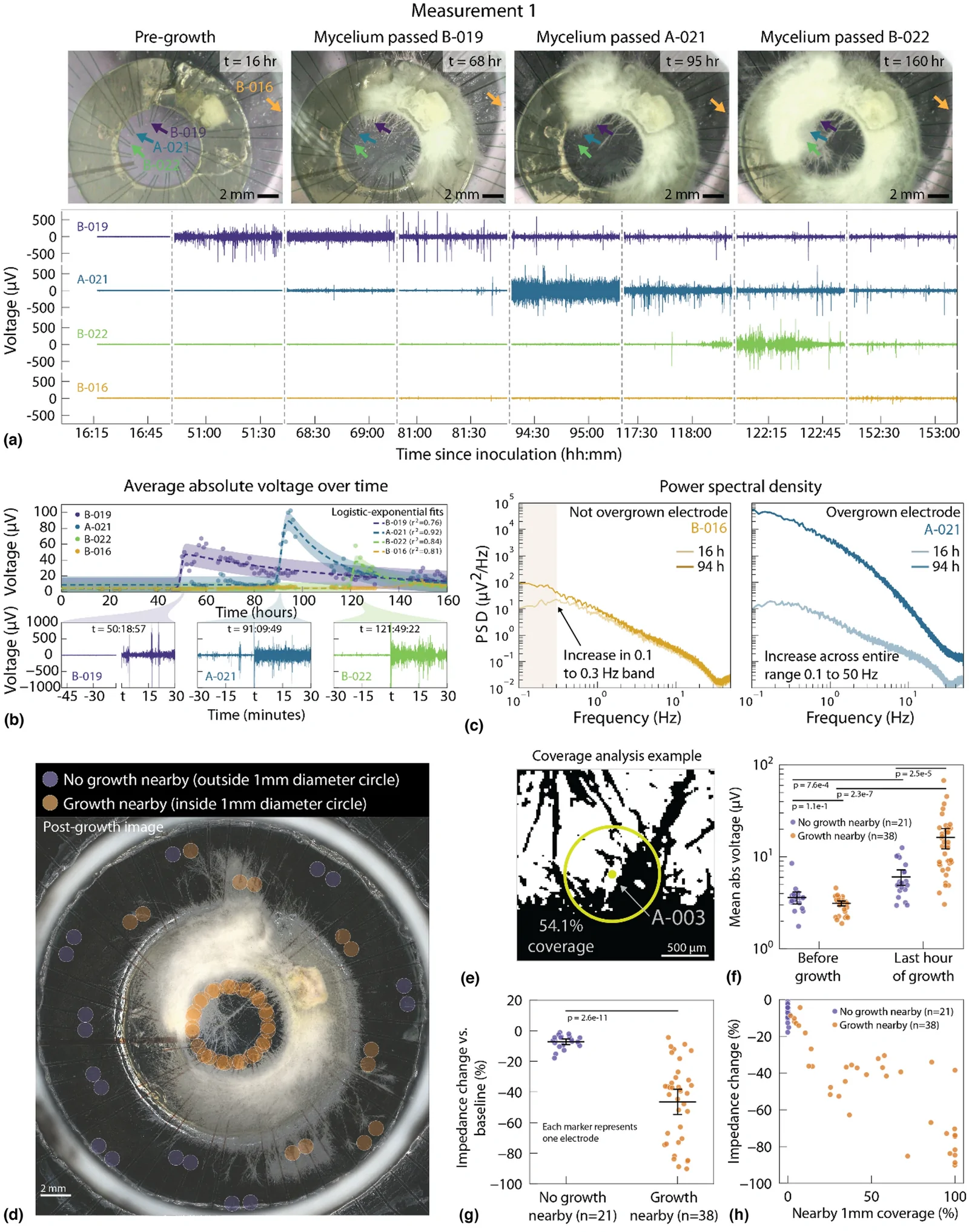
Fungal growth and the appearance of the electrical signal in Measurement 1. (a) *Top* images of the mycelium growth during the experiment, with key electrodes marked. B-019, A-021, and B-022 were chosen to represent the evolution of the signal as mycelium grew across the MEA, while B-016 was chosen as a representative electrode without mycelium growth. *Bottom* voltage traces from eight recorded 1-h segments as mycelium grows in the vicinity of the electrodes. (b) *Top* mean absolute voltage of each channel over time, with the data binned into 1-h segments, from hour 0; the inoculation time. The data are fitted with descriptive logistic-rise/exponential-decay functions that illustrate how the voltage rapidly increases and then slowly decays. *Bottom* voltage over time for the segments of data near the rising phase in the absolute average voltage data. (c) Comparison of the power spectral density plots for a channel without growth nearby (B-016) and an overgrown channel (A-021), before growth and during the period of highest activity for A-021. (d) The final growth pattern of the mycelium, 159 h after inoculation, × 38 magnification, stitched image. The image is overlaid with 1 mm diameter circular regions centred on each electrode, with colour depending on whether mycelium is present within the area (*n* = 59 electrodes). (e) An example image of how area coverage analysis was performed, depicting a 1 mm circular area centred on electrode A-003. Mycelium appears as black, and background as white. (f) The mean absolute voltage of all electrodes from Measurement 1, before growth (hour 16, *n* = 59) vs. end of growth (hour 159, *n* = 59). The mean and 95% confidence intervals are overlaid on the data, and *p*-values for Welch’s *t*-test difference of means are displayed for relevant groupings. (g) The impedance change from the end of growth compared to the pre-growth baseline for electrodes grouped by “No growth nearby” (*n* = 21) and “Growth nearby” (*n* = 38). The mean and 95% confidence intervals are overlaid on the data, and the *p*-value for Welch’s *t*-test difference of means is displayed. (h) The impedance change from the end of growth compared to the pre-growth baseline for *n* = 59 electrodes, plotted as impedance change vs. area coverage, determined by the method depicted in (e).

To record extracellular potentials during mycelial growth, we inoculated a custom microelectrode array (MEA; Fig. S1) with *G. lucidum* mycelium and monitored electrical activity over 160 h. Recordings were acquired over the growth period as near-continuous long-duration recordings, with occasional gaps caused by software interruptions and subsequent restarts. For analysis, the available data were divided into 1-h segments (see “Methods: Hourly voltage quantification” in the Supplementary Information) to compare electrical activity across different stages of colonisation. To focus the discussion, Fig. 2(a) shows eight representative 1-h analysis segments extracted from the available recording data for four selected electrodes positioned at increasing distances from the inoculum (full dataset available at https://doi.org/10.4121/1776a3f5-b539-4abe-85fe-ffddf5cc26a3). We chose B-019, A-021, and B-022 because the growth front crossed these electrodes in sequence, which can be used to correlate the growth and the signal. We used B-016 as a comparison electrode because mycelium did not appear to grow nearby. The signal without mycelial growth contains no spikes and mostly consists of *a* ± 10 µV noise floor on all channels, consistent with the expected noise level of the Intan amplifier chips. The uninoculated MEA control showed a similar baseline and no spike-like activity beyond this level (Fig. S2). Mechanical perturbation tests further confirmed that vibration-induced artefacts were readily distinguishable from the slower voltage spikes analysed here (Video S1).

Over the remainder of the experiment, the mycelium grew across the agar ring and onto the MEA, eventually overgrowing many electrodes [Fig. 2(a)]. By 160 h, the reference electrode was also overgrown, introducing common-mode signals into the recordings. Because these signals appeared broadly across channels with near-identical amplitude, we subtracted the median of all channels at each time point from each recording channel, thereby reducing the shared common-mode component. This robust common-average referencing (CAR) reduces signals that are simultaneously present across many channels and therefore limits their contribution to subsequent peak detection and spatial analyses. However, genuinely widespread biological signals may also be attenuated by this procedure, so the CAR-referenced data should be interpreted primarily as emphasising spatially local differences between electrodes. The effect of this processing is illustrated for three randomly selected 10-min segments in Figs. S3, S4, and S5, each showing five randomly selected channels. In most cases, the common-mode signal contains only sparse, low-amplitude (< 100 µV) peaks. This signal is present in all other channels and is readily removed once the median signal is subtracted. The correlation matrices in Figs. S6 and S7 likewise show that inter-channel correlation decreased after processing, consistent with the removal of shared common-mode activity. There is still some residual correlation, which may reflect close-by electrodes measuring similar signals from the growing mycelium on the MEA.

An important variable to understand the effect of is humidity and condensation. In our measurements, humidity was carefully controlled by tightly sealing the growing chamber on the MEA with vacuum-sealing tacky tape, including the lid and all holes used for sensor placement. Additionally, a small compartment in the growing chamber was used to store 1 mL of sterile, warm distilled water inside the sealed growing environment. There were no indications of leaks, as the humidity measurement inside the chamber showed humidity consistently above 92% for the entire measurement (Fig. S8). The same sealing method was used for all measurements and controls. This high humidity level led to visible condensation on the MEA throughout the measurements, but the absence of spike-like activity before mycelial growth in Fig. 2(a) (when humidity was already above 92% and condensation was already clearly present) and in an uninoculated control (Fig. S2) suggests that condensation and humidity did not produce the observed electrical behaviour.

In this measurement, electrical activity increased sequentially across the selected electrodes as the mycelium expanded. Electrodes B-019, A-021, and B-022 showed transitions from baseline noise level to larger voltage fluctuations in the same order as the advancing growth front, whereas B-016 remained comparatively quiet throughout most of the experiment [Fig. 2(a)]. On electrodes in proximity to the growth front, activity increased as mycelium approached or covered the electrode and then decreased after the active front had passed, consistent with stronger extracellular activity near actively growing regions of the colony. A similar increase-and-decay pattern was reported previously in needle electrode measurements.^[8]^ On shorter timescales, the recordings contained discrete extracellular voltage spikes with variable amplitudes, typically ranging from approximately 500 to 2000 µV in the examples shown (Fig. S9). These spikes occurred as both positive and negative deflections, were often biphasic, and developed over timescales of seconds.

To quantify how electrical activity evolved over time, we calculated the mean absolute voltage for each 1-h segment, which represents a rough measure of activity, accounting for biphasic and oppositely signed electrical signals. In the electrodes shown in the upper row of Fig. 2(b), activity increased rapidly over several hours as the advancing mycelium reached the electrode region, followed by a slower decay over subsequent days. To describe this temporal pattern, we fitted the data with a descriptive logistic-rise/exponential-decay function, which gave *R*^2^ values of 0.76 to 0.92. The rapid increase may correspond to the arrival of the active growth front near the electrode, while the decay phase may reflect the front moving beyond the electrode and leaving a more mature mycelial network behind.

The lower row of Fig. 2(b) shows 1-h recordings near the onset of the rapid increase in mean absolute voltage. During this period, the increase in activity appears to arise from changes in both spike occurrence and amplitude. These changes may correspond to local growth events, such as hyphae approaching or contacting the electrode. In Fig. 2(b) (bottom, middle), spikes are already present before the larger transition in activity, suggesting that there is not a discrete moment that spikes begin, but rather the activity level simply increases as the mycelium grows. The presence of discrete transitions may relate to more discrete events, such as contact with an electrode, which may cause acute changes to the electrical interface. However, higher-resolution imaging would be required to determine the true spatial relationship between individual hyphae and proximate electrodes.

Because the growth-associated spikes were relatively slow, we next tested whether their spectral power was concentrated in the low-frequency range reported in previous fungal recordings. Figure 2(c) compares power spectral density (PSD) estimates from a high-activity electrode near visible mycelial growth (A-021) and a comparatively low-activity electrode that remained farther from growth for the entire experiment (B-016). The analysis was performed over 0.1–50 Hz, corresponding to the usable frequency range of the 100 Hz down-sampled dataset. Higher frequencies were not analysed because 20 kHz recordings showed substantially less signal power above 50 Hz (Fig. S10). On A-021, mycelial growth was accompanied by a marked increase in spectral power, most prominently below 10 Hz, consistent with previous fungal recordings using microelectrodes (1.5–8 Hz),^[15]^ ITO glass (0.1–1 Hz),^[22]^ and needle electrodes (0.001–1 Hz).^[9]^ In contrast, B-016 showed only a smaller increase, concentrated between 0.1 and 0.3 Hz. In this dataset, the 0.3 to 10 Hz range may relate more closely to local mycelial growth, while lower frequencies could be measured even without the direct presence of mycelium at the electrode.

To track how the spectral content changed over time, we calculated bandpower in three frequency ranges for A-021 (Fig. S11). The pattern of increase and decrease in the bandpower closely followed that of the average absolute voltage in the low, middle, and high bandpower range. The noticeable exception was the 0.1–1 Hz band increased around 60 h, peaking before the larger broadband increase observed around 95 h. This earlier low-frequency increase suggests that electrical activity associated with nearby mycelial growth may become detectable before the onset of the larger-amplitude broadband fluctuations observed later in the experiment.

To test whether electrical activity corresponded to mycelium presence across the full MEA, we grouped the recording electrodes according to their final spatial relationship to the visible mycelial colony after 159 h of growth. By this time, the mycelium had expanded around the agar ring and across the MEA, with substantial variation in biomass quantity and morphology near each of the 59 recording electrodes [Fig. 2(d)]. For classification, we centred a 1 mm diameter circle on the electrode location and converted the corresponding image region to binary to distinguish mycelium from background. We then calculated the percentage of the circular area covered by visible mycelium. Electrodes with less than 1% coverage were classified as “No growth nearby”, whereas all remaining electrodes were classified as “Growth nearby”. An example of this analysis is shown in Fig. 2(e). Because imaging was performed from above, this classification cannot distinguish direct contact with the electrode from hyphae passing above it, and it therefore represents the general proximity of visible biomass rather than confirmed electrode contact. The electrodes assigned to each group are listed in Table S1.

Using this proximity-based categorisation, we compared the mean absolute voltage across all electrodes in Measurement 1 before growth (hour 16) and at the end of the experiment [hour 159, Fig. 2(f)]. Before growth, all but one electrode (B-006) had a mean absolute voltage between 1 and 5 µV, with no significant difference between the “No growth nearby” and “Growth nearby” groups (3.62 vs. 3.12 µV; Welch’s *t*-test, *p* = 0.11), consistent with a similar low-noise baseline across the MEA. By hour 159, the mean absolute voltage had increased in both groups, reaching 6.1 µV for electrodes with no growth nearby and 16.3 µV for electrodes with growth nearby. The “Growth nearby” group showed substantial variability, consistent with differences in the extent and progression of mycelial growth across the array. Despite this variability, mean absolute voltage remained significantly higher in the “Growth nearby” group (Welch’s *t*-test, *p* = 2.5 × 10^−5^). These results show that electrodes located near visible mycelium exhibited greater electrical activity than electrodes without visible growth nearby. This spatial association supports a relationship between increased electrical activity and mycelial growth, rather than a uniformly distributed measurement artefact or noise.

Another indicator of mycelial presence is a change in electrode impedance. Using the recording system’s on-board impedance measurement function, we measured impedance at several stages of growth. Figure 2(g) compares the impedance change from baseline to the end of the experiment. Electrodes with no growth nearby showed only a small decrease in impedance (mean − 7.2%), whereas electrodes with growth nearby showed a much larger decrease (mean − 46.4%), with a significant difference in mean between the groups (Welch’s *t*-test, *p* = 2.6 × 10^−11^). This suggests that the decrease in impedance is associated with the presence of nearby mycelial biomass. This may reflect changes in the local ionic and hydration environment around the electrode, which could lower impedance relative to the bare MEA interface. This interpretation is consistent with previous reports that mycelium can conduct electrical signals and form conductive bridges across otherwise insulating gaps.^[24,25]^ Zhao et al.^[26]^ further demonstrated frequency- and distance-dependent electrical signal attenuation in mycelial networks and showed that signal transmission is strongly influenced by hydration state. These observations reinforce that the impedance measured here likely depends not only on the quantity of nearby biomass, but also on its hydration, connectivity, and conductive pathway to the electrode.

The magnitude of the impedance decrease varied substantially among electrodes with nearby growth. Figure 2(h) shows that greater visible area coverage tended to be associated with a larger impedance decrease, although this relationship was weak. This variability suggests that additional factors, such as local biomass density/volume, hydration, or whether direct contact with the electrode is established, may influence the impedance change. Because these factors could not be resolved from the top-down images, their contributions could not be evaluated here. In the future experiments, the use of transparent substrates, higher-magnification imaging, and topographical imaging of the mycelium would help reveal more detailed insights relating biomass quantity and proximity to electrical activity.

**Figure 3:**
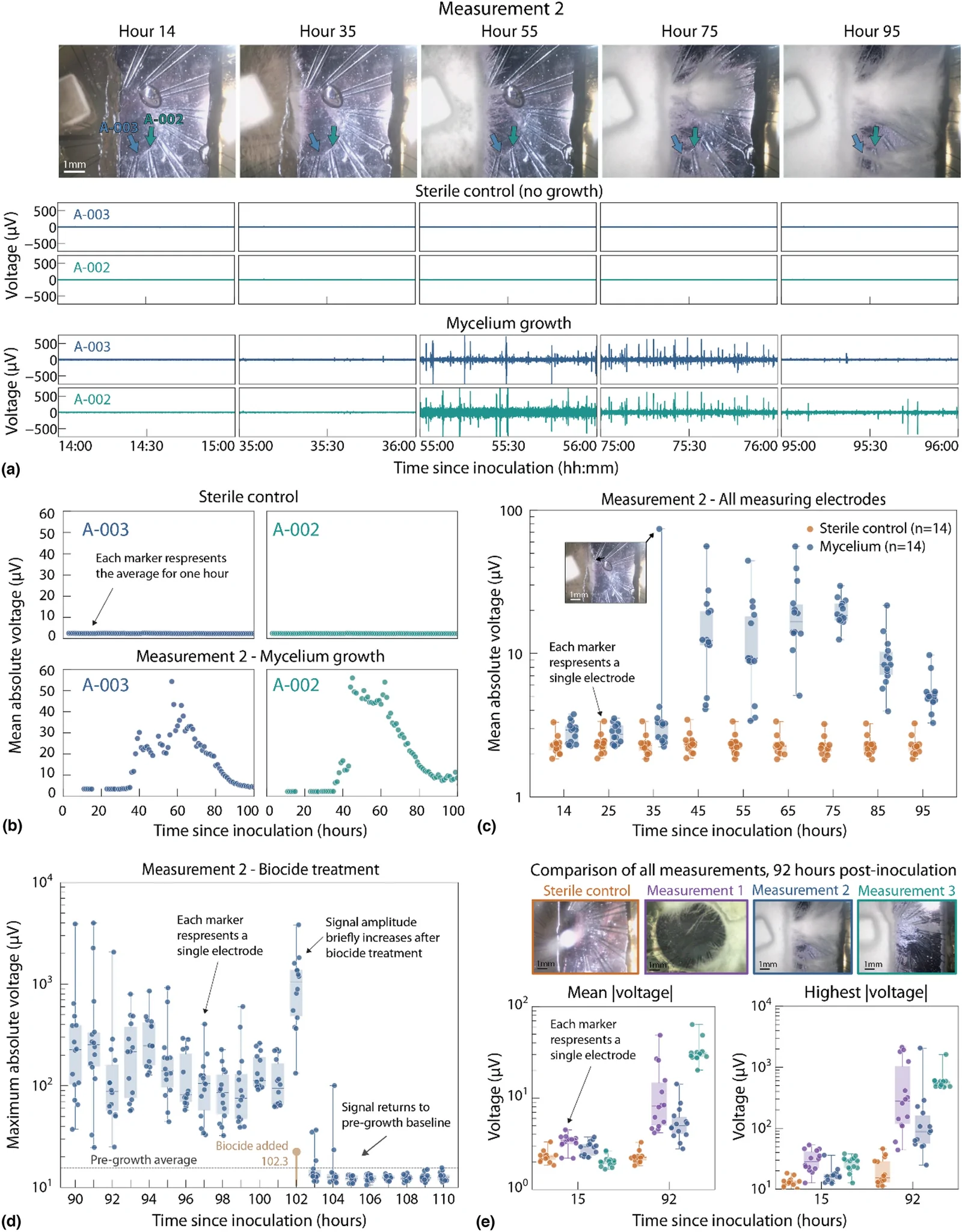
Additional measurements of fungal electrical activity. (a) *Top* images of the mycelium growth during Measurement 2 at evenly spaced hours from inoculation, with two featured electrodes A-002 and A-003 marked. *Bottom* voltage traces from the same 1-h segments for both a sterile control (no mycelium growth), and the mycelium measurement. (b) The mean absolute voltage over time, binned in 1-h segments, for Measurement 2 compared to a sterile control over the same duration for electrodes A-002 and A-003. (c) The mean absolute voltage over time for all measuring electrodes within the field of view, for both Measurement 2 and the sterile control. Box plots are overlaid on the figure, representing the median, interquartile range, and minimum and maximum of the data in each group. An inset plot highlights the electrode at the time corresponding to the data point depicted by the arrow. (d) The maximum of the absolute value of the voltage, binned into 1-h segments from hours 90 to 110 after inoculation. The pre-growth average and time of biocide addition are depicted. (e) *Top* images of each measurement, including the sterile control, at 92 h post-inoculation. *Bottom*, *left* the mean absolute voltage at hour 15 vs. hour 92, comparing all measurements. Box plots are overlaid on the figure, representing the median, interquartile range, and minimum and maximum of the data in each group. *Bottom*, *right* the maximum absolute voltage at hour 15 vs. hour 92, comparing all measurements.

To test whether the growth-associated electrical activity observed in Measurement 1 was reproducible under a modified experimental configuration, we performed a second measurement using a new but identical MEA, a new recording device, and a different agar arrangement designed to produce a horizontal, directional growth pattern. The results in Fig. 3(a) show that, as in Measurement 1, the mycelium progressively grew across the MEA. Growth was faster, reaching the analysed electrode region within approximately 95 h rather than 160 h because of the shorter gap between the nutrient-containing agar slabs. The left agar slab was also positioned over the reference electrode to reduce reference-associated common-mode signals. The same data processing steps were used prior to analysis as for Measurement 1.

The voltage traces in Fig. 3(a) show that two representative electrodes from the inoculated MEA followed a similar temporal pattern to Measurement 1. The electrical activity increased during the active growth phase and decreased as the local mycelium matured. The same electrodes in a sterile control prepared and measured under the same conditions showed no comparable increase in voltage. The hourly mean absolute voltage traces in Fig. 3(b) further demonstrate the transient increase in activity in the inoculated measurement and the stable baseline in the sterile control, providing confidence in the mycelium origin of the signal. Figure 3(c) shows that this effect extended across all recording electrodes within the field of view. Whereas the sterile control remained comparatively stable throughout the measurement, the inoculated MEA showed increases in mean absolute voltage across the analysed electrodes, with substantial variability consistent with differences in local growth progression and electrode proximity. The inset image in Fig. 3(c) shows how the electrode with the highest average voltage was also the one closest to the active growth front at that time.

To further test the association between mycelial activity and the recorded electrical signals, we applied a biocide treatment at the end of Measurement 2. Following the addition of 5% sodium hypochlorite solution to the agar slab containing the inoculum, the signal amplitudes initially increased and then returned to approximately the pre-growth baseline within 2 h [Fig. 3(d)]. These observations are consistent with the experimental framework proposed by Buffi et al.,^[15]^ who combined shielded differential electrode recordings, uninoculated controls, frequency-domain analysis, and chemical perturbations to distinguish fungal-associated activity from environmental and electrode-related artefacts. In their experiments, cycloheximide treatment strongly reduced the measured frequency content, whereas the carrier control did not. Our MEA measurements complement this approach by providing spatially distributed recordings that can be compared with the optically tracked progression of the mycelial growth front. Together, these approaches emphasise the importance of combining spatial measurements with appropriate controls and perturbations when interpreting spontaneous fungal electrical activity.

To assess whether the observed growth-associated activity was repeatable, we performed a replicate of Measurement 2, termed Measurement 3. Mycelial growth was slower in Measurement 3, such that the colony remained in an active growth phase at hour 92, whereas growth in Measurement 2 had progressed further by the same time point. Figure 3(e) compares all three measurements and the sterile control at hour 92. Despite differences in growth progression, mean and maximum absolute voltage increased from hours 15 to 92 in each inoculated measurement, while the sterile control remained near baseline. Across the measurements performed here, increased electrical activity was therefore consistently associated with growing mycelium.

**Figure 4:**
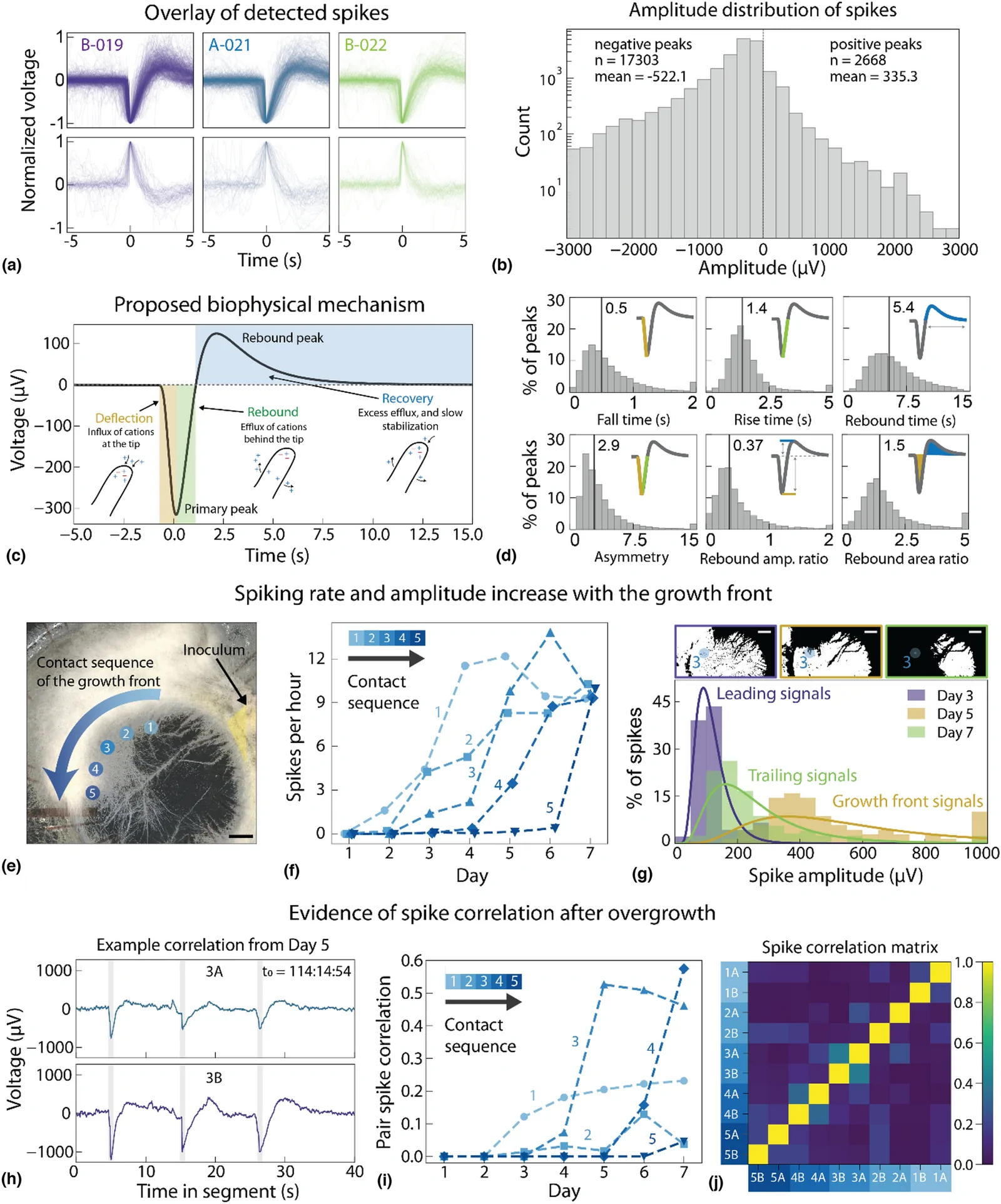
Fungal voltage spike characterisation and analysis. (a) Overlay plots of the spikes detected from the same select channels from Fig. 2 using a prominence threshold of 5 times the noise level (*σ*) and amplitude threshold of 10*σ*, separated into negative and positive spikes. (b) The distribution of spike amplitudes, with a log *y*-axis scale. The histogram is divided into 30 bins, and the rightmost and leftmost bins represent overflow bins. (c) The proposed ion-based signalling mechanism to explain spikes similar to action potentials in growing mycelium. (d) Distributions of the fitted spike characteristics, including fall time (s), rise time (s), recovery time (s), peak asymmetry, rebound amplitude ratio, and rebound area ratio. The vertical line represents the median value of the distribution. Each histogram is divided into 20 bins, and the rightmost bin represents an overflow bin. The inset graphics describe each parameter. (e) The relation of spiking rate and amplitude with the growing front of the mycelium. The labels 1, 2, 3, 4, and 5 each represent their respective pairs of channels on the MEA. The direction of the growth front is indicated by the arrow, showing sequential overgrowth of electrodes 1–5. Scale bar in the bottom right represents 1 mm. (f) The rate of spiking per day of the experiment. Spikes per hour are calculated by dividing all detected spikes for that electrode pair by the number of measurement hours for that pair on that day. (g) The distribution of spike amplitude on days 3, 5, and 7 for channel pair 3, with lognormal fits. The distribution is split into 20 bins, with an overflow bin at 1000 µV. The images above depict the binarised image of the growth front on those days, with channel pair 3 labelled, scale bar represents 1 mm. (h) An example of a 40-s segment of signalling data on day 5 demonstrating correlated spikes between the two electrodes from electrode pair 3. (i) The electrode pairs correlation by experiment day. (j) The spike correlation matrix, depicting the correlation of spike timing across channels.

Having observed growth-associated extracellular electrical activity across all three measurements, we next quantified the discrete voltage spikes detected across the MEA with a focus on data from Measurement 1. Spikes were detected across all 59 recording channels using a conservative amplitude threshold of 10*σ* and prominence threshold of 5*σ*, where *σ* was estimated from the local robust standard deviation of each signal (Fig. S12). This threshold was chosen to focus the analysis on high signal-to-noise events. Overlays of spikes from other channels also showed consistent waveforms across the MEA (Fig. S13). Amplitude-normalised overlays revealed predominantly biphasic spikes, with a rapid negative primary deflection followed by a slower rebound and recovery [Fig. 4(a), top], as well as a smaller population of positive spikes with analogous timing and biphasic shape [Fig. 4(a), bottom]. Across all detected spikes on all 59 electrodes, negative spikes were more common than positive spikes, with 17,303 negative spikes and 2668 positive spikes detected [Fig. 4(b)]. Spike amplitudes ranged from approximately − 3000 to 3000 µV, although most spikes were below 500 µV in absolute amplitude.

The detected spikes share some waveform features with extracellular recordings of action potential-like events. This characteristic has been noted in prior research, leading to the use of action potential-like terminology to describe the extracellular voltage spikes of mycelium.^[8,9]^ However, action potentials are a specific type of propagating electrical signal typically associated with neurons that relate to a triggered response of a cell to external stimuli by the active transport of ions through gated ion channels.^[27]^ The use of this term should be reserved for signals with clear biological signalling origin. In our experiments, we did not identify a signalling mechanism responsible for these spikes, so we instead refer to them as extracellular voltage spikes. Despite this, it is interesting to note their similarities to action potentials [illustrated in Fig. 4(c)], regarding the initial deflection, rebound, and recovery phases, which resemble the depolarisation, repolarisation, and hyperpolarisation phases of an action potential.^[28]^ In these phases of an action potential, there is a brief rise and fall in membrane potential, as ions enter and leave the cell, which was confirmed extracellularly using a microelectrode array.^[29]^

Action potentials are also not exclusive to neurons, as action potential-like signals have been measured in plants, specifically in electrically active plants such as the Venus flytrap (*Dionaea muscipula*) or the sensitive plant (*Mimosa pudica*).^[30]^ These signals are typically longer in duration, 3–20 s for plants vs. 1–2 ms for neuronal APs.^[30]^ Quantification of the detected spikes in our measurements showed characteristic timescales of seconds rather than the milliseconds associated with neuronal action potentials [Fig. 4(d)]. The median fall time, rise time, and recovery time were 0.5 s, 1.4 s, and 5.4 s, respectively. The median peak asymmetry, defined as the ratio of the fall time to the rise time, was 2.9. This indicates that the recovery period was considerably slower than the initial deflection, which is a typical feature of biological signals like action potentials. The median rebound amplitude ratio was 0.37, and the median recovery area ratio was 1.5, indicating that the secondary phase made up a substantial part of the waveform. This biphasic nature could be explained by a prolonged stabilisation period after the large initial spike, the biological origin of which is unclear. Interestingly, this same biphasic behaviour has been observed in both needle electrode and MEA recordings of growing mycelium.^[8,17,22]^

We then asked how spike activity changed as the mycelial growth front progressed across the array. Five electrode pairs located along the visible growth trajectory were analysed in the order in which the growth front reached their projected locations [Fig. 4(e)]. Spike rate increased sequentially across these electrode pairs, following the same spatial order as colony expansion [Fig. 4(f)]. For each pair, the spike rate rose over several days as visible mycelium developed near the electrodes, then reached a plateau of approximately 9–10 spikes per hour. The period of increasing spike rate before the plateau typically lasted two to three days, corresponding to the time during which more hyphal tips grow and develop the mature mycelium network. This pattern supports the interpretation that spike activity increases as the local mycelial network develops near the recording electrodes.

Spike amplitude also changed with growth-front progression as shown in Fig. 4(g). For electrode pair 3 (highlighted in the inset), low-amplitude (100–200 µV) spikes dominated on day 3, when visible growth was still approaching the electrode region. By day 5, when the growth front overlapped the projected electrode location, the amplitude distribution shifted towards larger events, with many spikes exceeding 500 µV. By day 7, after the leading growth front had moved beyond the electrode region, the distribution shifted again towards lower amplitudes. This temporal shift is consistent with a transition from weak detection of nearby activity to stronger activity during local growth-front passage, and then to residual activity from the more mature mycelial network behind the front.

Finally, we checked whether any temporal relationship between spikes developed alongside the formation of the dense mycelial network. The electrodes 3 A and 3B from Fig. 4(e) on day 5 showed aligned spikes over a 40 s recording segment [Fig. 4(h)]. We calculated spike correlation as the proportion of spikes on one electrode that occurred within ± 1 s of a spike on the paired electrode. Across the five electrode pairs, spike correlation increased after visible growth reached the corresponding electrode regions, capping out at approximately 50 to 60% spike correlation [Fig. 4(i and j)]. These correlations suggest that nearby electrodes can detect shared activity within a connected local (within 500 µm) mycelial network, although further experiments will be required to determine whether this is due to active biological propagation.

The spike analysis shows that the growth-associated electrical activity observed across the MEA consists of discrete extracellular voltage spikes with recurring waveform features, characteristic timescales, and growth-dependent changes in rate and amplitude. The increase in spike rate, the shift in amplitude distributions, and the emergence of time-aligned spikes in densely colonised regions support the idea that the extracellular potential fluctuations are linked to local mycelial network development. Even though these measurements alone do not establish the underlying biological mechanism, their association with mycelial growth suggests a biological, or at least mycelium-caused origin. In addition, the measurements provide quantified features such as spike rate, amplitude, waveform shape, spectral content, and spatial correlation that can be compared across future measurements.

## Conclusions

Our measurements showed an association of *G. lucidum* growth with the spontaneous emergence of extracellular electrical activity. This activity appeared as discrete voltage spikes with recurring biphasic waveforms, second-scale dynamics, and growth-dependent changes in rate and amplitude. Using spatially distributed microelectrodes, we found that electrical activity increased as the visible mycelial growth front approached individual electrodes and decreased after the active front had passed, indicating that regions near the advancing growth front were associated with stronger local electrical activity. The MEA setup, together with the shielded recording, controls, and post-processing, helped distinguish growth-associated voltage spikes from baseline electrical noise and experimentally induced artefacts. By quantifying spike waveform parameters, frequency content, impedance changes, and spatial correlations, this work provides a framework for comparing fungal extracellular voltage signals across growth stages, morphologies, and future experiments. Our findings are broadly consistent with prior needle electrode measurements, while adding spatially resolved information about local growth-associated activity. Overall, this work supports the view of mycelium as a living network exhibiting growth-associated extracellular electrical activity and provides a basis for future studies of fungal bioelectricity in sensing and functional living materials.

## Supplementary Information

Below is the link to the electronic supplementary material.

Supplementary file1 (DOCX 4677 kb) Supplementary file2 (MP4 42724 kb)

## Data Availability

The data that support the findings of this study are openly available at https://doi.org/10.4121/1776a3f5-b539-4abe-85fe-ffddf5cc26a3.
