# Supplementary material for: Microelectrode array measurements of fungal mycelium reveal growth-associated electrical activity
Source: MRS Commun. 2026 Sep 28;16(5):1481–91. doi: 10.1557/s43579-026-01041-7 (PMC13743589; doi:10.1557/s43579-026-01041-7)
Supplement: Supplementary file 1 [file 43579_2026_1041_MOESM1_ESM.docx]

**Supplementary Information: Microelectrode array measurements of fungal mycelium reveal growth-associated electrical activity**

Mark Ablonczy^1^, Sourav Patranabish^1,2^, Sarah Schyck^1^, Saurav Sharma^1^, Hande Aydoğmus-Sasse^2^, Vasso Giagka^3^, Clementine Viellard-Boutry^2^, Kunal Masania^1*^

^1^Shaping Matter Lab, Faculty of Aerospace Engineering, Delft University of Technology, Kluyverweg 1, 2629 HS Delft, Netherlands

^2^Electronic Components, Technology and Materials (ECTM), Department of Microelectronics, Faculty of Electrical Engineering, Mathematics and Computer Science, Delft University of Technology, Mekelweg 4, 2628 CD Delft, Netherlands

^3^Bioelectronics, Department of Microelectronics, Faculty of Electrical Engineering, Mathematics and Computer Science, Delft University of Technology, Mekelweg 4, 2628 CD Delft, Netherlands

*Corresponding author: [k.masania@tudelft.nl](mailto:k.masania@tudelft.nl)

**Contents:**

Materials and methods.

**Figure S1.** Microelectrode array layout

**Figure S2.** Noise floor measurement of uninoculated MEA

**Figure S3.** Robust CAR (median) signal subtraction – Ex 1

**Figure S4.** Robust CAR (median) signal subtraction – Ex 2

**Figure S5.** Robust CAR (median) signal subtraction – Ex 3

**Figure S6.** Example one-hour segment correlation before CAR-referencing

**Figure S7.** Example one-hour segment correlation after CAR-referencing

**Figure S8.** Humidity and temperature through the experiment

**Figure S9.** Example spikes from Measurement 1

**Figure S10.** PSD of a sample of high-frequency (20 kHz) measurement during growth

**Figure S11.** Example of bandpower change over time from Measurement 1

**Figure S12.** Peak detection method

**Figure S13.** Signal overlays from all MEA channels

**Figure S14.** Custom MEA adapter board

**Table S1.** Mycelium growth categorisation and area coverage

**Video S1.** Induced mechanical noise effect on measurement

**Materials and methods**

**Microelectrode array fabrication**

A 1µm SiO₂ layer was deposited by Plasma Enhanced Chemical Vapor Deposition (PECVD) on a 500µm-thick 4-inch silicon wafer. After this step, a Ti/TiN layer (20nm/300nm) was sputtered, followed by lithography and dry etching. Lithography consisted of photoresist spin coating, exposure with a mask aligner to define the electrodes, and development. Dry etching was performed in an Inductively Coupled Plasma Reactive Ion Etching (ICP-RIE) machine using a chlorine-based chemistry. O₂ plasma and HNO₃ were used to remove organic residues from the surface and as cleaning steps. Then, a UV-sensitive polyimide layer was spin-coated, patterned and hard-baked in an N₂ oven at 200 °C to form the insulation layer, leaving only the electrode sensing areas and contact pads without polyimide. Finally, a blade dicer was used to remove the peripheral silicon area from the chip. An SLA-printed (White resin V4.1) ring (28 mm inner diameter) was adhered directly to the MEA using cyanoacrylate glue. The MEA was then thoroughly cleaned using a three-step procedure of Acetone, Isopropyl alcohol, and distilled water. Three identical MEAs were fabricated together, and of those, two were used for the measurements described in this manuscript. MEA 1 was used for Measurement 1, and MEA 2 was used for Measurement 2, 3, and the sterile control, while being cleaned and sterilised with 70% ethanol between uses.

**Electrophysiology recording setup – Measurement 1**

The device used to record the signals was an [Intan RHD USB Interface Board](https://intantech.com/RHD_USB_interface_board.html). This device supports sampling rates from 1 kS/s to 30 kS/s and can be connected to a host computer by USB cable. Two [Intan RHD2132 amplifier chips](https://intantech.com/products_RHD2000.html) were connected to the MEA through a custom adapter board (fabricated by our collaborator at the Fraunhofer Institute) (Figure S14). The adapter board was connected to the MEA's contact pads via onboard pogo pins that compressed to make contact. The MEA was aligned and fixed in place with the adapter board using a custom 3D-printed stage. This stage was then attached to a steel base plate through vibration-damping feet. The entire assembly was then placed into a container of sand to further isolate it from vibrations, and then inside a large Faraday shield (50 cm x 50 cm x 50 cm) made of 2 mm thick steel plating, which was connected to ground through the Intan board’s GND pin, as suggested by the manufacturer. Signals were saved to a host computer placed outside of the Faraday shield, along with the Intan interface board. The Intan RHX software was used to collect signals from the MEA at 1 kHz. A single, short 60 s recording at 20 kHz was also collected during the active growth phase of the mycelium. Amplifier bandwidth was set to 0.13 to 100 Hz. Recordings were acquired over the 160 h growth period as near-continuous long-duration measurements. Occasional gaps occurred when the recording software stopped unexpectedly. In these cases, the recording was restarted as soon as possible. The available recordings were aligned to the experiment start time and divided into one-hour segments for analysis. Before recording Measurement 1, mechanical artefacts were characterised by tapping/banging the side of the Faraday cage, and moving and twisting cables and external components (laptop, tethers, etc) to determine the effect of mechanical disturbance on the recorded signal. These artefacts left only brief ( less than 1 s) high frequency, low amplitude sinusoidal signals, that were easily distinguished from signals associated with mycelial growth.

**Electrophysiology recording setup – Measurements 2, 3, and Sterile control**

The device used to record the signals was an [Open Ephys Acquisition Board (3^rd^ Generation).](https://open-ephys.org/acquisition-system/oeps-9029) This device supports sampling rates from 1 kS/s to 30 kS/s and can be connected to a host computer by USB cable. Two [Intan RHD2132 amplifier chips](https://intantech.com/products_RHD2000.html) (identical to those used for Measurement 1, but newly purchased) were connected to the MEA through the same custom adapter board as Measurement 1 (**Figure S14**). The assembly method, sampling rate (1 kHz), and amplifier bandwidth (0.13–100 Hz) were identical to those of Measurement 1. Recordings were acquired over a shorter, roughly 100-hour growth period, so more measurements could be taken rapidly. Recordings were continuous except for occasional gaps when the recording software stopped unexpectedly. In these cases, the recording was restarted as soon as possible. The available recordings were aligned to the experiment start time and divided into one-hour segments for analysis.

**Mycelium culturing**

The medium for mycelium growth was malt extract agar, consisting of 100 g/L malt extract (Fisher Scientific), 15 g/L agar (Carl Roth), 5 g/L peptone (Merck Sigma). The malt extract agar was sterilised at 121 °C for 20 minutes using an autoclave (Hirayama HG-50). A culture of *Ganoderma lucidum* (strain M9726, originally from a grain spawn bag from Mycelia NV) was grown on malt extract agar in a petri dish for 7 days at 27 °C, then stored in the fridge at 9°C until further use.

**Inoculation – Measurement 1**

An agar ring was prepared by casting still-liquid malt extract agar into an 18 mm (outer) x 8 mm (inner) x 1.25 mm SLA-printed sterile mould. The microelectrode and adhered ring were sterilised by soaking in 70% ethanol for 20 minutes. Immediately after, the agar ring was placed directly onto the microelectrode array, working inside a biosafety cabinet (Herasafe) to ensure sterility. A rectangle of 4 mm x 4 mm was cut from the pre-grown *G. lucidum* culture and transferred to the agar ring on the microelectrode array. The microelectrode array adapter and the lid of the containment ring were assembled in the biosafety cabinet, after which the entire assembly was transferred to the Faraday shield.

**inoculation – Measurements 2, 3, and Sterile control**

Two semi-circular slabs of agar, 15 mm in diameter were prepared by cutting them out of a pre-cast sterile petri dish of malt-extract agar. The microelectrode and adhered ring were sterilised by soaking in 70% ethanol for 20 minutes. Immediately after, the agar slabs were placed on the microelectrode array, working inside a biosafety cabinet (Herasafe) to ensure sterility. The slabs were positioned at an approximate distance of 5 mm apart near the center of the MEA, so that one of the slabs was directly on top of the reference electrode. A rectangle of 4 mm x 4 mm was cut from the pre-grown *G. lucidum* culture and transferred to the left agar slab (over the reference) on the microelectrode array. The microelectrode array adapter and the lid of the containment ring were assembled in the biosafety cabinet, after which the entire assembly was transferred to the Faraday shield.

**Growth monitoring**

Humidity and temperature inside the MEA container were monitored using an embedded Adafruit Sensirion SHT40 (Product ID: 4885, Adafruit Industries) connected to an Arduino Mega 2560 (SKU A000067, Arduino) inside the Faraday shield. The sampling rate of the sensor was set to one sample per minute. The noise from the Arduino sampling was a single tens-of-ms-duration monophasic spike, which was readily removed from the signal during post-processing. To maintain humidity above 95%, sterile distilled water was added to a small side well on the container at the start of the experiment, and all gaps in the container were sealed with adhesive sealant (Tacky Tape SM5126, Amrize). Growth was optically tracked using a USB microscope (AM7915MZTL, Dino-Lite US) with integrated LED lighting, positioned above the MEA, which remotely captured one timelapse image per hour. A 3-day (Measurement 1 setup) and later a full 7-day (Measurement 2 setup) uninoculated control measurement were also collected.

**Biocide control**

Biocide treatment was administered at the end of Measurement 2, at 102 hours after inoculation. The biocide was 5% sodium hypochlorite solution (lab cleaning bleach). While the measurement was paused, a sterile syringe and needle was carefully used to pierce the lid of the growing chamber and deposit three droplets directly onto the mycelium biomass on the agar slab on the left. Afterwards, the pierced lid was re-sealed, and the recording was immediately continued for another 48 hours. The humidity and temperature remained at the same level as before biocide addition.

**Data preprocessing**

Raw voltage data from the 59 channels was exported as minute-by-minute segments in RHD files, and then converted into collated HDF5 files for each continuous segment of data. The HDF5 files were downsampled by 10 compared to the original RHD files to increase workability of the dataset, resulting in a sample rate of 100 Hz and a Nyquist frequency of 50 Hz. These files were then converted into a long-format parquet table containing 100 Hz data for each channel with timestamps aligned to the experiment start time. A robust common-average reference (CAR) was computed as the sample-wise median across all available channels and subtracted from each channel to reduce common-mode noise. The CAR-subtracted traces were subsequently smoothed with a centred 3-sample rolling median filter, to remove high-frequency events such as Arduino spikes, without affecting longer-duration events. Short artefact intervals relating to external noise, such as opening the Faraday shield door, were then flagged using predefined experiment-hour windows (hour 47: 3100-3125 s, hour 87: 3300-3330 s, and hour 156: 3000-3100 s within the hour). These samples were marked with an artefact mask. The processing steps were identical for all Measurements, except that raw data for measurements with the Open Ephys system was in DAT files instead of RHD.

**Hourly voltage quantification**

The processed signals were aggregated by channel and hour since inoculation after excluding masked samples. Within each channel-hour bin, the mean absolute voltage, mean signed voltage, standard deviation, maximum absolute voltage, and the number of retained samples was computed. Hourly bins with partial data due to measurement interruptions were also utilised in the analysis, given that at least 10 minutes of data was available. For each channel, all non-missing hourly values were plotted as individual datapoints over time, and the temporal trajectory was modelled with a one-peak logistic-rise-plus-exponential-decay function shown below,

$$V\left( t \right)\text{=}\alpha\text{+}A\text{*}\frac{1}{1\text{+}exp\left( \text{-}\frac{t\text{-}c}{b} \right)}\text{*}exp\left( \text{-}kmax\left( t\text{-}c,0 \right) \right)$$

where V(t) is the voltage at time t, α is the baseline voltage, A is the rise amplitude, c is the rise-centre time, b is the rise width, and k is the exponential decay rate. In this model, voltage was expressed as a baseline term plus a sigmoidal rise that transitioned into exponential decay after the rise centre. To visualise uncertainty around each fitted trajectory, we calculated an approximate 95% prediction band as the fitted curve plus or minus 1.96 times the residual standard deviation for that channel, with the lower bound clipped at 0 µV. The functions were fit to each channel of data by nonlinear least squares regression using SciPy’s curve_fit function.

Group comparisons of mean absolute voltage of all electrodes grouped by “Growth nearby” (n=38) or “No growth nearby” (n=21) were performed using Welch’s t-test for a difference of sample means. P-values are reported without adjustment for multiple comparisons because the analyses consisted of a limited number of predefined comparisons addressing distinct hypotheses. The results should therefore be interpreted in the context of the individual comparisons rather than as a single family-wise inference. Data are reported as means with 95% confidence intervals. These tests were performed on electrode-level observations within a single culture and should not be interpreted as evidence of reproducibility across independent biological replicates.

**Frequency-domain analysis**

The power spectral density (PSD) was estimated with Welch’s method (scipy.signal.welch) using a Hann window, constant detrending, a sampling frequency of 100 Hz, and nperseg = 8192 samples (or the full available trace if shorter), with 50% overlap (noverlap = nperseg/2). Only positive frequencies were retained. The resulting PSD curves were plotted on log-log axes from 0.1 to 50 Hz. For the bandpower series, one PSD was computed per hour using the same method described above. The PSD was then averaged within three frequency bands (0.1-1 Hz, 1-5 Hz, and 5-50 Hz) to produce an hourly band-power time series. The same logistic-exponential functions were fitted as those described in the hourly voltage quantification section.

**Classification of mycelial growth**

After the full 160 hours of mycelial growth, the Measurement 1 MEA was imaged using a Keyence VR-5000 Profilometer Stitching Microscope. An image of the entire MEA was captured at 39 x magnification by image stitching. Mycelial growth was classified into two groups: no growth nearby, and growth nearby. The stitched 39 x magnified post-growth image was binarized by thresholding in ImageJ Fiji at a grayscale value of 80, then using the denoise function once. For each electrode, a 1 mm diameter circle was drawn at the centre of the electrode, then the area that was black (mycelium) was measured as % area of that circle. If the % area was less than or equal to 1%, the electrode was categorised as “No growth nearby”, if it was greater than 1%, it was classified as “Growth nearby”. The full list of electrode classifications for Measurement 1 is shown in **Table S*1***, and is available in the repository. Growth classification was performed without reference (blinded) to the electrophysiological data.

**Impedance measurements**

Impedance was measured using the Intan RHX software at 1, 5, 10, 50, and 100 Hz. Measurements were taken before, at various stages during, and after growth. Impedance change was quantified as the percentage change in impedance at 10 Hz between measurements during growth and a measurement taken at 16 hours, before any growth occurred. Group comparisons of impedance change of all electrodes grouped by “Growth nearby” (n=38) or “No growth nearby” (n=21) were performed using Welch’s t-test for a difference of sample means. Impedance measurements were acquired from the same 59 electrodes recording electrophysiology data on the MEA. Impedance was measured at discrete time points and was not performed simultaneously with the long-duration voltage recordings. When impedance was probed, the ongoing electrophysiology recording was interrupted for approximately 5 minutes and then resumed on the same channels. Due to the low currents (on the order of pico- to nano-amperes) and short probing times, no artefacts or long-term effects on the signal were noticeably present.

**Replicate and control voltage analysis**

Measurement 2, Measurement 3, and the sterile control were processed using the same preprocessing and hourly aggregation procedures as for Measurement 1. Experiment time was aligned to inoculation, with 00:00 corresponding to the time of inoculation for each measurement. For comparisons across measurements, mean absolute voltage and maximum absolute voltage were calculated for each recording electrode in one-hour bins.

For the array-wide comparison shown in Figure 3c, all working recording electrodes visible within the optical field of view were included. Hourly mean absolute voltage values were compared across the inoculated measurement and sterile control to assess whether growth-associated changes were observed across the analysed electrode region.

For the cross-measurement comparison shown in **Figure 3e**, electrode-level mean absolute voltage and maximum absolute voltage were compared at hour 15, representing the pre-growth baseline, and hour 92, representing a later stage of mycelial development. These time points were used to compare the change in electrical activity between Measurement 1, Measurement 2, Measurement 3, and the sterile control while retaining a common inoculation-relative time point.

**Spike detection**

For spike detection, we operated on the processed voltage traces after robust common-average referencing, median filtering, and artefact masking. Peaks were detected separately for positive and negative deflections using scipy.signal.find_peaks applied to the signal. Detection thresholds were defined relative to a local noise estimate computed independently for each channel per hour as a robust standard deviation (sigma) derived from the median absolute deviation as

$$MAD\text{=}median\left( \left| x_{i}\text{-}median\left( x \right) \right| \right)$$

$$\sigma_{robust}\text{=}1.4826\text{*}MAD$$

Spikes were detected using a prominence threshold of 5σ and a minimum absolute amplitude of 10σ, with a minimum peak-to-peak spacing of 20 ms. The detection thresholds were intentionally selected to prioritise high signal-to-noise events rather than maximise the number of detected spikes. The 10σ minimum-amplitude criterion reduces the inclusion of low-amplitude fluctuations that may arise from baseline noise, and the 5σ prominence criterion requires detected spikes to be distinct from other local fluctuations. For Gaussian-distributed background noise, excursions beyond ±10σ would be vanishingly rare (P(|X| > 10σ) ~1.5 × 10⁻²³), making the 10σ minimum-amplitude criterion highly conservative with respect to ordinary baseline noise. We note, however, that non-Gaussian artefacts can exceed such thresholds and were addressed separately by use of common-average referencing. Spike onset and offset were defined by expanding outward from the detected peak until the absolute signal dropped below 1σ. To avoid counting biphasic spikes as two separate spikes, any event of opposite sign occurring within 5 s of the previous retained event on the same channel was excluded.

**Spike analysis**

Spike characteristics were analysed by first modelling the spikes with a representative function. Each detected spike was fitted with a biphasic two-lognormal waveform, with a baseline as the median signal in the 0.5 s before the start of the peak event. The fitted waveform was the sum of two lognormal-shaped components:

$$V\left( t \right)\text{=}V_{0}\text{+}A_{1}L\left( t;{\text{-}m}_{1},m_{1,\text{ }}\sigma_{1} \right)\text{+ }A_{2}L\left( t;o_{2},m_{2,\text{ }}\sigma_{2} \right)$$

$$L\left( t;o,\text{ }m,\text{ }\sigma\right)\text{= }\left\{ \begin{aligned} 0,\text{ }t\text{ }\leq o \\ {exp\left( \frac{\text{-}\left[ ln\left( t\text{-}o \right)\text{-}\left( lnm\text{+}\sigma^{2} \right) \right]^{2}}{2\sigma^{2}} \right)}/{exp\left( \frac{{\text{-}\sigma}^{2}}{2} \right)},\text{ }t\text{>}o \end{aligned} \right.$$

where V_0_ is the baseline voltage, A_1_ & A_2_ are the amplitudes of the first and second phase, m_1_ & m_2_ are the mode parameters, σ_1_ & σ_2_ are the shape parameters and o_2_ is the onset time of the second phase. This function is derived from the standard lognormal probability density function

$$f\left( x \right)\text{= }\frac{1}{x\sigma\sqrt{2\pi}}exp\left( \text{-}\frac{{\text{ }\left( lnx\text{-}\mu\right)}^{2}}{2\sigma^{2}} \right),\text{ }x\text{>}0$$

where the variable is shifted from x to time after onset:

$$x\text{=}t\text{-}o$$

parametrisation is changed from *µ* to the mode *m,* and the peak occurs at *x = m*:

$$x_{mode}\text{=}m\text{=}exp\left( \mu\text{- }\sigma^{2} \right)$$

$$\mu\text{= }lnm\text{+ }\sigma^{2}$$

and the function is peak-normalised so that the amplitude parameter directly equals the peak size by dividing by the exponential term when *x = m*:

$$exp\left( \text{-}\frac{\sigma^{2}}{2} \right)$$

The prefactor $1\text{/}(x\sigma\sqrt{2\pi})\text{ }$is not needed, and is omitted.

Parameters were estimated by nonlinear least squares using SciPy’s curve_fit, minimising the sum of squared residuals between the raw spike and the model waveform. Initial values were set to the detected peak and the largest opposite-polarity peak after the first peak, and parameter bounds were imposed to facilitate faster convergence. After fitting, the goodness-of-fit was summarised using R^2^. The fitted parameters were then used to derive summary features, including the amplitudes of the first and second phases, their amplitude and area ratios, fall time, rise time, and rebound time. For all analyses, only events with successful fits and R^2^ > 0.9 were retained, to ensure that the fitted waveforms accurately represented the underlying data.

**Spatial spike analysis**

To examine how spike activity changed with the progression of the mycelial growth front, five electrode pairs located along the visible growth trajectory in Measurement 1 were selected and analysed in the order in which the growth front reached their projected electrode regions. Spike rate was calculated for each electrode pair and experiment day by dividing the total number of detected spikes by the total number of recorded hours available for that pair on that day.

For the analysis of spike amplitude distributions, detected spikes from electrode pair 3 were grouped by experiment day. Amplitude distributions were compared for days 3, 5, and 7, corresponding to stages when visible mycelial growth was approaching, overlapping, and had progressed beyond the projected electrode region, respectively.

Temporal coincidence between spikes on nearby electrodes was quantified using a ±1 s coincidence window. For a given electrode pair, spike correlation was calculated as the proportion of spikes on one electrode that occurred within ±1 s of a spike on the paired electrode. This analysis was repeated across the selected electrode pairs and experiment days to evaluate how temporal coincidence changed as the local mycelial network developed. A spike-correlation matrix was generated using the same coincidence criterion to compare spike timing across channels.

**Microelectrode array layout**

**Figure S1.** The microelectrode array (MEA 1), used for Measurement 1, with each channel label next to its corresponding contact pad. The electrodes are circular 100 µm diameter exposed regions at the ends of each channel’s trace.

**Noise floor measurement of uninoculated MEA**

**Figure S2.** A 60-minute segment of data from hour 20 of the control measurement on MEA 1 for Measurement 1, where the agar ring was present, but without mycelium inoculum. The y-axis range for each subplot is -200 to 200 µV. The lack of extracellular voltage signals due to the lack of fungal growth is evident here. Channels A-030 and A-029 have higher baseline noise levels than the other electrodes due to improper contact of the adapter board pogo pins with the MEA contact pads. On all other channels, the noise level remains around ± 10 µV.

**Robust CAR (median) signal subtraction – Ex 1**

**Figure S3.** An example 10-minute segment of data from hour 158 from Measurement 1 for a random selection of five channels, demonstrating the CAR common-mode referencing method. **(Top)** The CAR reference trace, the median signal of all channels, with distinct spike events highlighted. **(Below)** The raw and CAR-subtracted trace of each of 5 randomly selected channels is shown, highlighting how the common-mode signals are present, and how it is removed after subtraction.

**Robust CAR (median) signal subtraction – Ex 2**

**Figure S4.** An example 10-minute segment of data from hour 113 from Measurement 1 for a random selection of five channels, demonstrating the CAR common-mode referencing method. **(Top)** The CAR reference trace, the median signal of all channels, with distinct spike events highlighted. **(Below)** The raw and CAR-subtracted trace of each of 5 randomly selected channels is shown, highlighting how the common-mode signals are present, and how it is removed after subtraction.

**Robust CAR (median) signal subtraction – Ex 3**

**Figure S5.** An example 10-minute segment of data from hour 148 from Measurement 1 for a random selection of five channels, demonstrating the CAR common-mode referencing method. **(Top)** The CAR reference trace, the median signal of all channels, with distinct spike events highlighted. **(Below)** The raw and CAR-subtracted trace of each of 5 randomly selected channels is shown, highlighting how the common-mode signals are present, and how they are modified by subtraction.

**Example one-hour segment correlation before CAR-referencing**

**Figure S6.** An example of Pearson correlation matrix for a one-hour segment, from hour 150, of data for all channel pairings of the MEA, for raw data before processing.

**Example one-hour segment correlation after CAR-referencing**

**Figure S7.** An example of Pearson correlation matrix for a one-hour segment, from hour 150, of data for all channel pairings of the MEA, for raw data after processing with 3-sample rolling median filter, and subtracting the median signal.

**Humidity and temperature through the experiment**

**Figure S8.** Humidity and temperature values recorded by the SHT40 inside the mycelium container on the MEA through the 160 h duration of Measurement 1.

**Example spikes from Measurement 1**

**Figure S9.** Example spikes recorded from featured channels, at various timestamps during Measurement 1.

**PSD of a sample of high-frequency (20 kHz) measurement during growth**

**Figure S10.** PSD plot of a 60-second segment of data measured at 20 kHz at hour 160 for channel A-021. The components of the signal above 50 Hz have substantially less power than the lower-frequency components.

**Example of bandpower change over time from Measurement 1**

**Figure S11.** Band power change over time for channel A-021from Measurement 1.

**Peak detection method**

**Figure S12.** An example of data from channel B-019, showing the local noise σ, and the absolute threshold 10σ.

**Signal overlays from all MEA channels**

**Figure S13.** The overlays shown here depict all detected signals across all channels with R^2^ greater than 0.9 from the lognormal fit.

**Custom MEA adapter board**

**Figure S14.**The custom printed circuit board, bottom view. The two amplifier chips connect to the connectors on the right side. The pogo pins in the centre compress into contact with the contact pads of the MEA.

**Table S1.** Mycelium growth categorisation and area coverage

| **Electrode** | **Group** | **% area coverage** | **Growth nearby?** |
| --- | --- | --- | --- |
| A-000 | inner | 4 | Growth nearby |
| A-001 | inner | 25.4 | Growth nearby |
| A-002 | inner | 25.6 | Growth nearby |
| A-003 | inner | 54.1 | Growth nearby |
| A-004 | inner | 38.2 | Growth nearby |
| A-005 | mid | 0 | No growth nearby |
| A-010 | inner | 59 | Growth nearby |
| A-011 | inner | 67.6 | Growth nearby |
| A-012 | mid | 0 | No growth nearby |
| A-013 | outer | 0 | No growth nearby |
| A-014 | mid | 71.7 | Growth nearby |
| A-015 | outer | 0.3 | No growth nearby |
| A-016 | mid | 0.3 | No growth nearby |
| A-017 | outer | 0 | No growth nearby |
| A-018 | mid | 3.9 | Growth nearby |
| A-019 | inner | 86.1 | Growth nearby |
| A-020 | inner | 99.9 | Growth nearby |
| A-021 | inner | 100 | Growth nearby |
| A-022 | inner | 100 | Growth nearby |
| A-023 | inner | 97 | Growth nearby |
| A-024 | mid | 0 | No growth nearby |
| A-025 | outer | 0 | No growth nearby |
| A-026 | mid | 14 | Growth nearby |
| A-027 | outer | 1.7 | Growth nearby |
| A-028 | mid | 85.6 | Growth nearby |
| A-029 | outer | 0.6 | No growth nearby |
| A-030 | inner | 47.1 | Growth nearby |
| A-031 | inner | 14.8 | Growth nearby |
| B-000 | inner | 10.5 | Growth nearby |
| B-001 | inner | 5 | Growth nearby |
| B-002 | inner | 12.6 | Growth nearby |
| B-003 | inner | 36.9 | Growth nearby |
| B-004 | inner | 56.7 | Growth nearby |
| B-005 | inner | 34.4 | Growth nearby |
| B-006 | outer | 0 | No growth nearby |
| B-007 | mid | 0 | No growth nearby |
| B-008 | inner | 57.8 | Growth nearby |
| B-009 | outer | 0 | No growth nearby |
| B-010 | mid | 0 | No growth nearby |
| B-011 | outer | 0 | No growth nearby |
| B-012 | mid | 80.2 | Growth nearby |
| B-014 | outer | 1 | No growth nearby |
| B-015 | mid | 6.6 | Growth nearby |
| B-016 | outer | 0 | No growth nearby |
| B-017 | mid | 0.3 | No growth nearby |
| B-018 | inner | 35 | Growth nearby |
| B-019 | inner | 95 | Growth nearby |
| B-020 | inner | 100 | Growth nearby |
| B-021 | inner | 100 | Growth nearby |
| B-022 | inner | 100 | Growth nearby |
| B-023 | inner | 100 | Growth nearby |
| B-024 | mid | 0 | No growth nearby |
| B-025 | outer | 0 | No growth nearby |
| B-026 | mid | 7.4 | Growth nearby |
| B-027 | outer | 0 | No growth nearby |
| B-028 | mid | 99.8 | Growth nearby |
| B-029 | outer | 0 | No growth nearby |
| B-030 | inner | 30.6 | Growth nearby |
| B-031 | inner | 41.5 | Growth nearby |
